# MKK3 modulates JNK-dependent cell migration and invasion

**DOI:** 10.1038/s41419-019-1350-6

**Published:** 2019-02-15

**Authors:** Yihao Sun, Di zhang, Xiaowei Guo, Wenzhe Li, Chenglin Li, Jingjing Luo, Mingcheng Zhou, Lei Xue

**Affiliations:** The First Rehabilitation Hospital of Shanghai, Shanghai Key Laboratory of Signaling and Diseases Research, School of Life Science and Technology, Tongji University, 1239 Siping Road, Shanghai, 200092 China

## Abstract

The c-Jun N-terminal kinase (JNK) pathway plays essential roles in regulating a variety of physiological processes including cell migration and invasion. To identify critical factors that regulate JNK-dependent cell migration, we carried out a genetic screen in *Drosophila* based on the loss-of-cell polarity-triggered cell migration in the wing epithelia, and identified *MKK3 licorne* (*lic*) as an essential regulator of JNK-mediated cell migration and invasion. We found that loss of *lic* suppressed *ptc* > *scrib-IR* or *ptc* > Egr triggered cell migration in the wing epithelia, and *Ras*^*v12*^*/lgl*^−/−^ induced tumor invasion in the eye discs. In addition, ectopic expression of Lic is sufficient to induce JNK-mediated but p38-independent cell migration, and cooperate with oncogenic Ras to promote tumor invasion. Consistently, Lic is able to activate JNK signaling by phosphorylating JNK, which up-regulates the matrix metalloproteinase MMP1 and integrin, characteristics of epithelial–mesenchymal transition (EMT). Moreover, *lic* is required for physiological JNK-mediate cell migration in thorax development. Finally, expression of human MKK3 in *Drosophila* is able to initiate JNK-mediated cell migration, cooperates with oncogenic Ras to trigger tumor invasion, and rescue loss-of-*lic* induced thorax closure defect. As previous studies suggest that MKK3 specifically phosphorylates and activates p38MAPK, our data provide the first in vivo evidence that MKK3 regulates JNK-dependent cell migration and invasion, a process evolutionarily conserved from flies to human.

## Introduction

About 90% of cancer patients die from tumor metastasis rather than primary tumor growth^1^. Therefore, finding effective ways to prevent or even reverse tumor cell invasion is of great significance to the treatment of cancer. To investigate the underlying genetic mechanisms, several invasion and metastasis models have been established in *Drosophila melanogaster*^2–4^. For example, depletion of cell polarity genes such as *scrib* along the anterior/posterior (A/P) compartment boundary in the wing epithelia produces an invasive cell migration phenotype^5^, while loss of cell polarity cooperates with oncogenic Ras (Ras^V12^) in the eye discs to promote tumor growth and invasion^6^. Previous work has identified the c-Jun N–terminal kinase (JNK) signaling as a crucial mediator of both invasive cell migration and tumor invasion in *Drosophila*^7–9^.

JNK belongs to the mitogen-activated protein kinase superfamily, and the JNK pathway plays crucial roles in many kinds of cellular behaviors, such as cell migration, proliferation, differentiation, apoptosis and stress reaction. The JNK pathway is highly conserved from *Drosophila* to human, while dysregulation of JNK signaling has been implicated in various human diseases, including cancer and neurodegenerative diseases^10,11^. Yet, it remains elusive how this pathway is tightly regulated in development, and factors that modulate this pathway have not been fully identified.

In mammalian cells, the p38 mitogen-activated protein kinase (MAPK) pathway is stimulated in response to a variety of environmental stresses and inflammatory stimuli. MKK3 is a protein kinase with dual specificity and belongs to the MAPK kinase family. Previous studies suggest that MKK3 specifically phosphorylates and activates p38 MAPK. In *Drosophila*, p38 MAPK is activated via dual phosphorylation at the Thr-Gly-Tyr motif by the MKK3 ortholog Licorne (Lic). For example, during oogenesis, Lic-p38 signaling is required in the germ line for correct asymmetric development of the egg^12^. Lic has also been shown to modulate other signaling pathways. For instance, Lic overexpression affects target gene expression of the Wingless (Wg) or Hippo pathway^13,14^. Consistently, MKK3 promotes nuclear localization of YAP via the actin cytoskeleton^14^. Despite all of the above, a role of MKK3 in JNK signaling has not been previously reported.

We have carried out a genetic screen in *Drosophila*, and found that *lic* is required for loss-of*-*cell polarity-triggered JNK-dependent invasive cell migration in the wing epithelia and oncogenic cooperation-induced JNK-mediated tumor invasion from the eye disc to the ventral nerve cord (VNC). In addition, ectopic expression of Lic activates JNK pathway by promoting JNK phosphorylation, triggers JNK-dependent invasive cell migration and cooperates with *Ras*^*V12*^ to promote tumor invasion. Moreover, *lic* is required for physiological JNK-mediated cell migration in thorax development. Furthermore, genetic epistasis analysis suggests that Lic acts in parallel with Hep as a potential JNK kinase. Finally, we found that expression of human *MKK3* in *Drosophila* also activates JNK signaling, triggers JNK-dependent cell migration, cooperates with oncogenic Ras to promote tumor invasion, and rescues loss-of-*lic* induced JNK-mediated thorax closure defect. Thus, we provide the first in vivo evidence that MKK3 regulates JNK-mediated cell migration and invasion, and this function of MKK3 is likely conserved from flies to human.

## Results

### *lic* is required for depletion of *scrib*-induced invasive cell migration

JNK signaling plays an important role in the regulation of cell migration and tumor invasion. Depletion of cell polarity genes, such as *scrib, dlg* and *lgl*, along the A/P compartment boundary of the *Drosophila* wing disc, triggers JNK-mediated invasive cell migration, a widely accepted in vivo model to study cell migration and invasion^15–17^.

To identify additional factors that regulate JNK-mediated cell migration and invasion, a candidate screen for dominant modifiers of *ptc* > *scrib-IR* induced invasive phenotype was carried out, in which the *ptc*-Gal4 driver was used to knock down *scrib* along the A/P boundary in the wing disc^18^. We have screened > 1000 *UAS-RNAi* lines from the Bloomington, Vienna *Drosophila RNAi* Center (VDRC) and National Institute of Genetics (NIG) stock centers targeting potential factors upstream of JNK, or factors that interact with JNK pathway components genetically or biochemically as reported in the literature. We have previously identified *Rho1*, *wnd*, *Src42*, *ben* and *dUev1* as modulators of JNK-mediated cell invasion from the screen^18–20^. The screen is still ongoing, as more RNAi lines are being added to the stock centers.

**Fig. 1:**
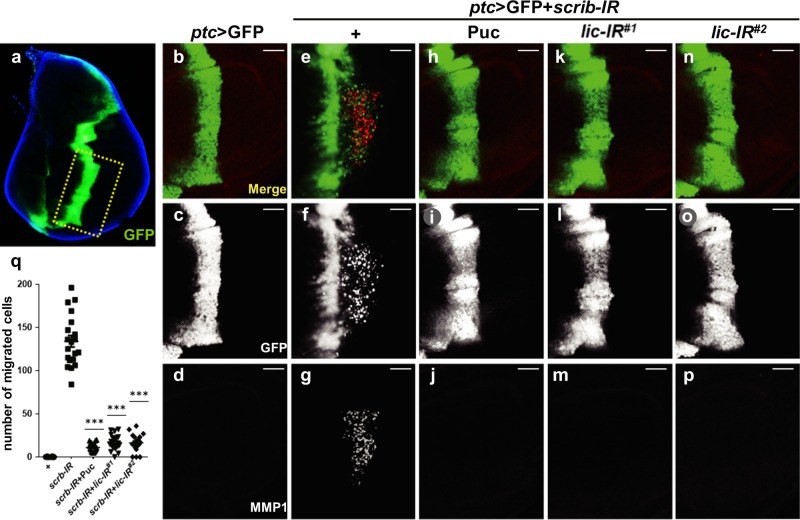
Lic is required for depletion of scrib-induced invasive cell migration in wing discs. Fluorescent micrographs of third instar wing discs (**a**–**p**) are shown. The dotted line depicts the area where cell migration occurs with *ptc* > GFP in the imaginal wing disc (**a**). *ptc*-Gal4 driven overexpression of *scrib-*RNAi promoted cells from A/P boundary migrating to posterior and MMP1 activation (**e**–**g**), compared to the GFP-expressing control (**b**–**d**). As a positive control, Puc blocked the cell migration phenotype and MMP1 staining (**h**–**j**). Expression of two independent *lic-RNAi* lines significantly inhibited cell invasion and MMP1 expression (**k**–**p**). Scale bars, 20 μm. The number of migrated cells were quantified and shown in (**q**), and one-way ANOVA test was used to calculate statistical significance, *n* ≥ 17, mean + s.d., ***P < 0.001

Compared with the *ptc* > GFP control (Fig. 1a–d), RNAi-mediated depletion of *scrib* resulted in a large number of cells delaminated from the A/P boundary and migrated to the posterior compartment (Fig. 1e, f), accompanied by the upregulation of MMP1 levels (Fig. 1g)^21^, which is one of the important molecular features of epithelial–mesenchymal transition (EMT)^22,23^. *puckered* (*puc)* is a transcriptional target of JNK signaling, but also encodes a JNK phosphatase that inhibits JNK activity^24,25^. As a positive control, Puc expression blocked *ptc* > *scrib-IR* induced cell migration and MMP1 activation (Fig. 1h–j), confirmed that both phenotypes depend on JNK signaling. To rule out the possibility of Gal4 titration by another UAS transgene, we used *UAS*-LacZ as a negative control, and confirmed that LacZ expression did not affect the cell migration phenotype (Supplementary Figure 1). From the screen, expression of two independent *lic-RNAi* lines were found to significantly inhibit depletion-of-*scrib* triggered cell migration and MMP1 upregulation (Fig. 1k–q). Furthermore, knockdown of *lic* significantly suppressed *scrib-IR*-induced JNK signaling activation (revealed by expression of the *TRE*-RFP and *puc*-LacZ reporters), JNK phosphorylation and accumulation of β-integrin (Supplementary Figure 2). The knockdown efficiencies of the *lic*-RNAi lines were verified by qRT-PCR (Supplementary Figure 3). Taken together, these data suggest that *lic* is physiologically required for JNK-dependent invasive cell migration triggered by loss of cell polarity.

### Lic promotes JNK-dependent cell migration

**Fig. 2:**
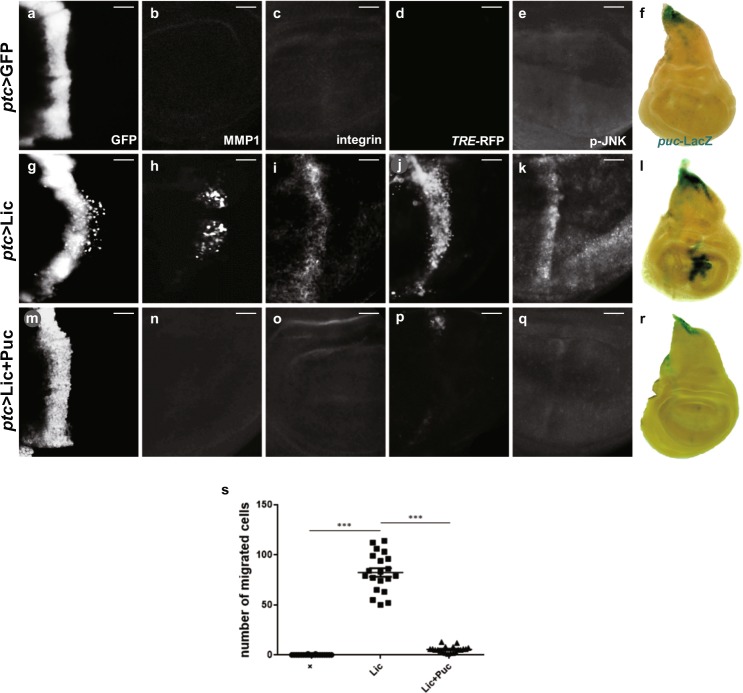
Lic promotes JNK-dependent cell invasion. Fluorescent (**a**–**e, g**–**k, m**–**q**) and light (**f, l, r**) micrographs of third instar wing discs are shown. Compared with the control (**a**–**f**), *ptc*-Gal4-driven overexpression of Lic induced cell migration (**g**), elevated expression of MMP1 (**h**), β-integrin (**i**), TRE-RFP (**j**), JNK phosphorylation (**k**) and *puc*-LacZ (**l**), which were inhibited by Puc expression (**m**–**r**). Scale bars, 20 μm. Statistics of migrated cell numbers was shown in (**s**), and One-way ANOVA test was used to calculate statistical significance, *n* ≥ 14, mean + s.d., ***P < 0.001

Next, we sought to test whether Lic promotes JNK-dependent cell migration in vivo. Compared with the *ptc* > GFP control (Fig. 2a–f), ectopic expression of Lic promotes some of the GFP-positive cells migrated to the posterior part (Fig. 2g, s), along with upregulated staining of MMP1 (Fig. 2h) and integrin (Fig. 2i), another biomarker for EMT^26,27^. Consistent with previous reports, both MMP1 and integrin were activated autonomously and non-autonomously^4^. Importantly, ectopic Lic-induced EMT-like invasive phenotypes were fully inhibited by the expression of Puc (Fig. 2m–o), or a dominant negative form of Bsk (Bsk^DN^) encoding the *Drosophila* JNK ortholog (Supplementary Figure 4).

These data suggest that JNK signaling is required for Lic-induced EMT-like cell invasion. To investigate whether Lic is sufficient to activate JNK signaling, we checked the expression of a *puc*-LacZ reporter, a commonly used readout of JNK signaling. Compared with the control (Fig. 2f), *puc*-LacZ expression was considerably activated along the A/P boundary by ectopic Lic expression driven by *ptc*-Gal4 (Fig. 2l), which could be significantly suppressed by co-expression of Puc (Fig. 2r). A *TRE*-RFP reporter containing four copies of an optimal Jun/Fos (AP-1) heterodimer binding site fused with RFP has been used to characterize the activation of JNK signaling^28^. Compared with the control (Fig. 2d), expression of Lic dramatically enhanced *TRE-*RFP expression (Fig. 2j), which was abolished by expressing Puc (Fig. 2p). Consistent with previous reports that JNK signaling propagates in the wing disc, Lic is able to activate JNK signaling both autonomously and non-autonomously (Supplementary Figure 5). Lic encodes the *Drosophila* MKK3 that regulates the p38 signaling in immunity^29^, oogenesis^12^, stress response^30^ and tissue growth^14,31^, yet its role in JNK signaling has not been previously reported. To investigate whether Lic is a kinase upstream of JNK, we checked JNK phosphorylation by a specific anti-p-JNK antibody^32^. We found that ectopic Lic was sufficient to promote JNK phosphorylation (Fig. 2k, q; Supplementary Figure 6). Thus, Lic is sufficient to activate JNK-dependent invasive cell migration in the wing disc. In contrast, expressing a kinase dead form of Lic (Lic^KD^) failed to produce any of the above phenotypes (Supplementary Figure 7), indicating that the kinase activity is required to induce invasive cell migration.

p38b has been proposed to play the central role in *Drosophila* p38 signaling^33–36^. However, Lic-induced JNK pathway activation and invasive cell migration were not affected by expressing a *p38b-RNAi* or a dominant-negative allele of p38b (p38b^DN^) (Supplementary Figure 8). Together, these data suggest that Lic activates the JNK signaling by promoting JNK phosphorylation in a mechanism independent of p38.

### Human MKK3 promotes JNK-dependent cell migration in *Drosophila*

**Fig. 3:**
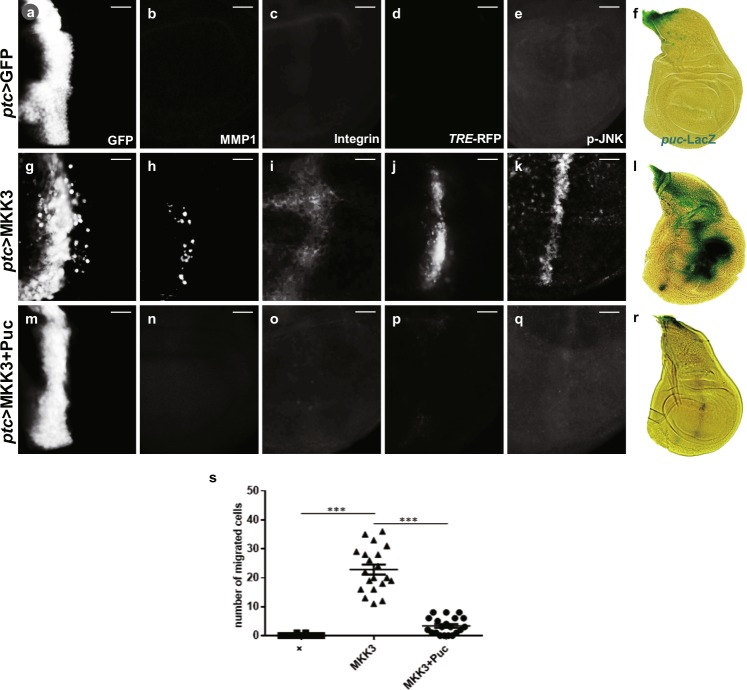
Expression of MKK3 induces JNK-dependent cell invasion in wing discs. Fluorescent (**a**–**e, g**–**k, m**–**q**) and light (**f, l, r**) micrographs of third instar wing discs are shown. Compared with the control (**a**–**f**), ectopic expression of MKK3 induced cell migration (**g**) accompanied by activation of MMP1 (**h**), integrin (**i**), *TRE-*RFP (**j**), p-JNK (**k**) and *puc*-LacZ (**l**), which were significantly suppressed by co-expression of Puc (**m**–**r**). Scale bars, 20 μm. Statistics of migrated cell numbers was shown in (**s**), and One-way ANOVA test was used to calculate statistical significance, *n* ≥ 14, mean + s.d., ***P < 0.001

Given that Lic encodes the *Drosophila* MKK3 ortholog and triggers JNK-dependent cell migration, it is of interest to know whether MMK3 retains Lic’s ability to promote JNK-mediated cell migration. A few in vitro studies have suggested that MKK3 is possibly involved in cancer cell invasion^37^, but this function of MKK3 has not been confirmed in vivo, and it remains unclear whether it is JNK dependent. To address this issue, a *UAS*-MKK3 transgene was generated and ectopically expressed the human MKK3 along the A/P boundary in the wing disc. Compared with the control (Fig. 3a–f), expression of MKK3 phenocopied that of Lic with induced cell migration (Fig. 3g, s), JNK phosphorylation (Fig. 3k), upregulation of MMP1, β-Integrin, *TRE*-RFP and *puc*-LacZ (Fig. 3h–j, l). Similar to Lic, these functions of MKK3 depend on JNK (Fig. 3m–r, Supplementary Figure 9), but not p38 (Supplementary Figure 10). Collectively, these data reveal that human MKK3 is functionally conserved with *Drosophila* Lic to promote JNK-dependent epithelia cell invasion in vivo, which is of great interest for future drug research targeting MKK3.

### Lic is required for *Ras*^*v12*^*/lgl*^−/−^-induced tumor invasion

**Fig. 4:**
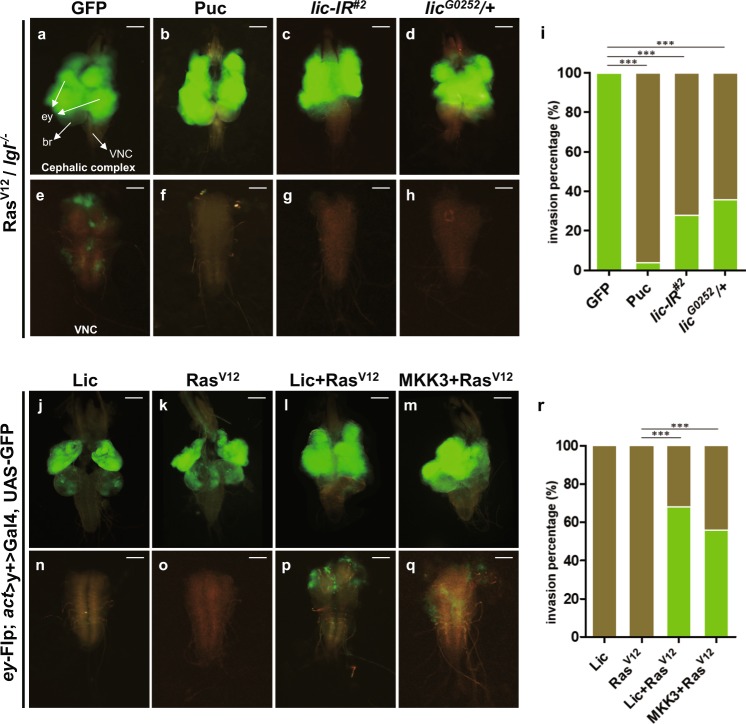
Lic is essential for oncogenic cooperation induced tumor growth and invasion. Dorsal views of the cephalic complexes (**a**–**d, j**–**m**) and VNCs (**e**–**h, n**–**q**) are shown. *Ras*^*V12*^/*lgl*^*−/−*^ induced tumor invasion into the VNC (**e**) was fully blocked by expression of Puc (**f**), and significantly suppressed by expressing a *lic-RNAi* (**g**) or in *lic* heterozygous mutants (**h**). A quantification of tumor invasion phenotype in **e**–**h** was shown in (**i**). One-way ANOVA test was used to calculate statistical significance, mean + s.d., n.s., *P* > 0.05; ***P < 0.001, and more than 30 samples were counted for each group. Eye disc clones expressing Lic (**j**) or Ras^V12^ (**k**) never invaded into VNC (**n**, **o**), while massive tumor growth and invasion to the VNC were observed by co-expression of Ras^V12^ and Lic (**l**, **p**), or Ras^V12^ and MKK3 (**m**, **q**). Scale bars, 200 μm in (**a**–**d**, **j**–**m**) and 100 μm in (**e**–**h**, **n**–**q**). A quantification of tumor invasion phenotype in **n**–**q** was shown in (**r**). One-way ANOVA test was used to calculate statistical significance, mean + s.d., ***P < 0.001, and more than 40 samples was counted for each group

Expression of oncogenic *Ras* is able to cooperate with loss of cell polarity, e.g. *lgl* mutation, in eye disc clones to promote tumor-like growth (Fig. 4a) and invasion into the ventral nerve cord (VNC) (Fig. 4e)^6,38^, a previously established in vivo model to study the mechanism of tumor invasion^19,32,39^. Consistent with previous data indicating that the JNK signaling plays a crucial role in *Ras*^*v12*^*/lgl*^−/−^ -induced tumor invasion^19,20^, expression of Puc completely blocked tumor cell invasion to VNC (Fig. 4f, i). We found that *Ras*^*v12*^*/lgl*^−/−^ -induced tumor invasion from eye to VNC was significantly inhibited by knocking-down *lic* or in heterozygous *lic* mutants (Fig. 4g–i). Intriguingly, the sizes of primary tumors in eye discs appeared to be not affected by JNK inactivation or loss of *lic* (Fig. 4b–d), implying Lic is specifically required for *Ras*^*v12*^*/lgl*^−/−^-triggered JNK-dependent tumor invasion.

### Lic/MKK3 synergizes with *Ras*^*V12*^ to promote tumor growth and invasion

Loss of cell polarity or activation of cell morphogenetic genes induces JNK-dependent invasive cell migration and synergizes with Ras^V12^ to initiate tumor growth and invasion^40,41^. Given that Lic triggers JNK-dependent invasive cell migration, we speculated that Lic might be able to cooperate with Ras^V12^ to promote tumor growth and invasion. Overexpression of Lic alone does not induce tumorigenesis or invasion (Fig. 4j, n), which is similar to that of Ras^V12^ (Fig. 4k, o)^6^. When Lic was simultaneously expressed with Ras^V12^, tumors formed from eye discs and invaded into the VNC region 8 days after egg laying (Fig. 4l, p, r), suggesting an oncogenic cooperation between Lic and Ras^V12^ is sufficient to promote tumor growth and invasion. Importantly, such synergistic effect also occurred between MKK3 and Ras^V12^ (Fig. 4m, q, r), suggesting that this function of Lic/MKK3 has been evolutionary conserved.

### Lic modulates JNK activity in parallel with Hep

**Fig. 5:**
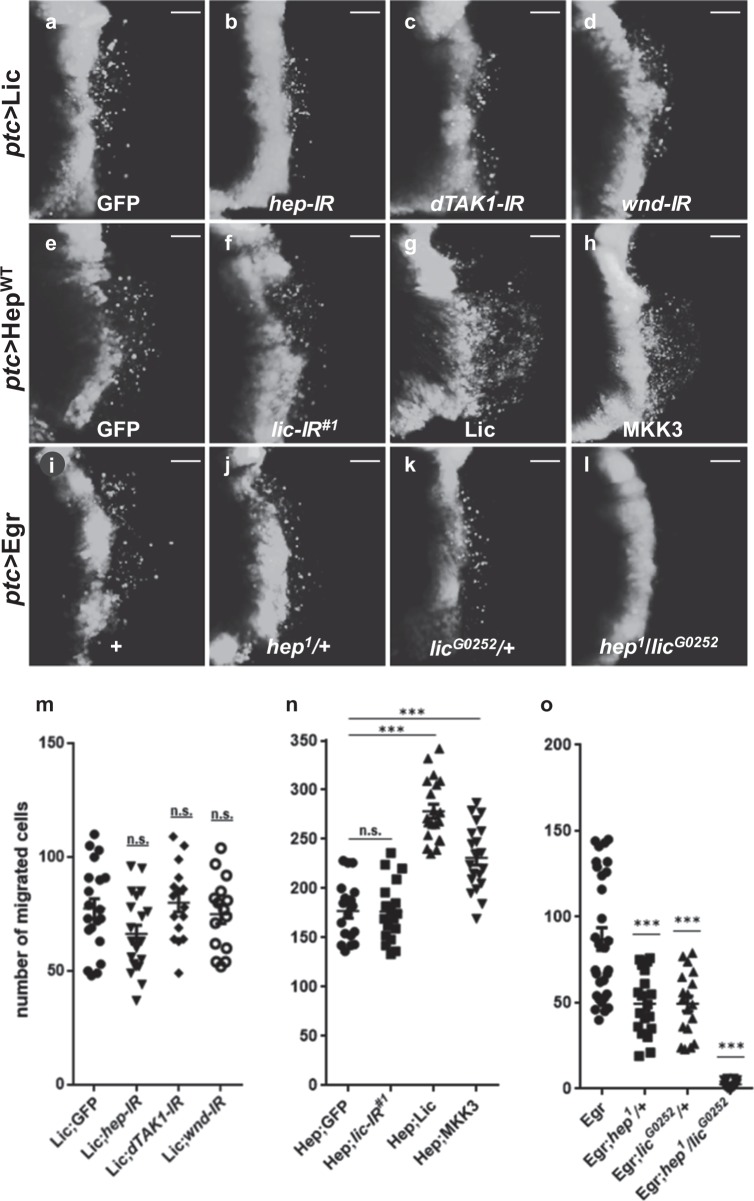
Lic and Hep act in parallel in the JNK pathway. Fluorescent micrographs of third instar wing discs (**a**–**l**) are shown. Compared with the GFP-expressing control (**a**), RNAi-mediated depletion of *hep*, *dTAK1* or *wnd* failed to suppress *ptc* > Lic induced cell invasion (**b**–**d**). *ptc*-Gal4 driven expression of Hep^WT^ induced a large number of cells migrating to the posterior of wing disc, which was not suppressed by expressing GFP (**e**) or *lic-RNAi* (**f**), but was synergistically enhanced by co-expressing Lic (**g**) or MKK3 (**h**). *ptc* > Egr induced cell migration (**i**) was partially inhibited in *hep* (**j**) or *lic* (**k**) heterozygous mutants, and fully blocked in their transheterozygous mutants (**l**). Scale bars, 20 μm. Statistics of migrated cell numbers was shown in (**m**–**o**), and One-way ANOVA test was used to calculate statistical significance, *n* ≥ 18, mean + s.d., n.s., *P* > 0.05; ***P < 0.001

Next, to investigate how Lic regulates JNK signaling, we performed epistasis analysis between Lic and JNK pathway components. As shown above, *ptc* > Lic-induced invasive cell migration and JNK activation were significantly inhibited by expressing Puc (Fig. 2m–s) or Bsk^DN^ (Supplementary Figure 4), suggesting that Lic acts upstream of Bsk. Next, we checked the genetic interaction between Lic and other kinases upstream of Bsk. Hemipterous (Hep) is a serine/threonine protein kinase involved in the JNK pathway by phosphorylating Bsk^42,43^. Both Lic and Hep belong to the mitogen-activated protein kinase kinase family (MAPKKs), while Wnd and dTAK1 are MAPKKKs that activate JNK via Hep activation. We found that knockdown of *hep*, *dTAK1* or *wnd* did not inhibit Lic-triggered cell migration (Fig. 5a-d, m and Supplementary Figure 11a–e). On the other hand, ectopic Hep-induced cell migration (Fig. 5e) was not suppressed by depleting *lic* (Fig. 5f), but was synergistically enhanced by co-expressing Lic or MKK3 (Fig. 5g, h, n). Egr encodes the *Drosophila* TNF ortholog that activates JNK-mediated cell migration (Fig. 5i), which was suppressed partially in heterozygous *hep* or *lic* mutants (Fig. 5j, k), but completely in transheterozygous mutants (Fig. 5l, o). Collectively, these data suggest that Lic likely activates JNK in parallel with Hep (Supplementary Figure 11f).

### Lic regulates physiological JNK-dependent thorax closure in development

**Fig. 6:**
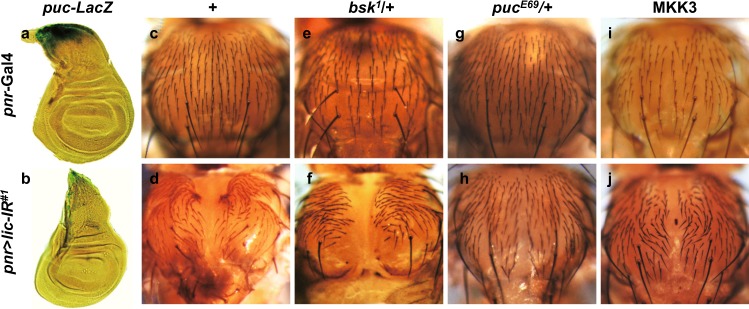
Lic regulates physiological JNK-mediated thorax closure. Light micrographs of *Drosophila* wing discs (**a**, **b**) and adult thoraxes (**c**–**j**) are shown. The endogenous *puc* expression pattern in the notum region of wing disc (**a**) was impeded by knockdown *lic* (**b**). Compared with the control thorax (**c**), loss of Lic-produced a thorax cleft phenotype (**d**), which was enhanced in heterozygous *bsk* mutants (**f**), and restored in heterozygous *puc* mutants (**h**), or by overexpression of MKK3 (**j**), whereas heterozygous mutation in *bsk* or *puc*, or expression of MKK3 gave no obvious phenotype (**e**, **g**, **i**, respectively)

Thorax closure serves as another in vivo model to study cell migration in *Drosophila* development. JNK signaling is crucial for thorax closure^44^, as reduced JNK activity results in a dorsal cleft phenotype in the thorax^45^. Consistent with this, endogenous JNK activation, indicated by *puc-*LacZ expression, was detected in the dorsal patch of the wing disc (Fig. 6a)^46^. RNAi mediated downregulation of *lic* under the control of *pannier* promoter (*pnr*-Gal4) abrogated *puc*-LacZ expression (Fig. 6b), and produced a thorax cleft (Fig. 6d) that phenocopies that of JNK inactivation^45^. The thorax defect was further enhanced in heterozygous *bsk* mutants (Fig. 6f), but was suppressed in heterozygous *puc* mutants (Fig. 6h) in which the endogenous JNK activity is increased^47^. Therefore, *lic* is required for physiological JNK-mediated cell migration in thorax closure. Intriguingly, depletion-of-*lic*-induced thorax defect was suppressed by expressing MKK3 (Fig. 6j), implying this function of Lic has been conserved in evolution as well.

### Lic-induced JNK-mediated cell invasion is not a consequence of cell death

**Fig. 7:**
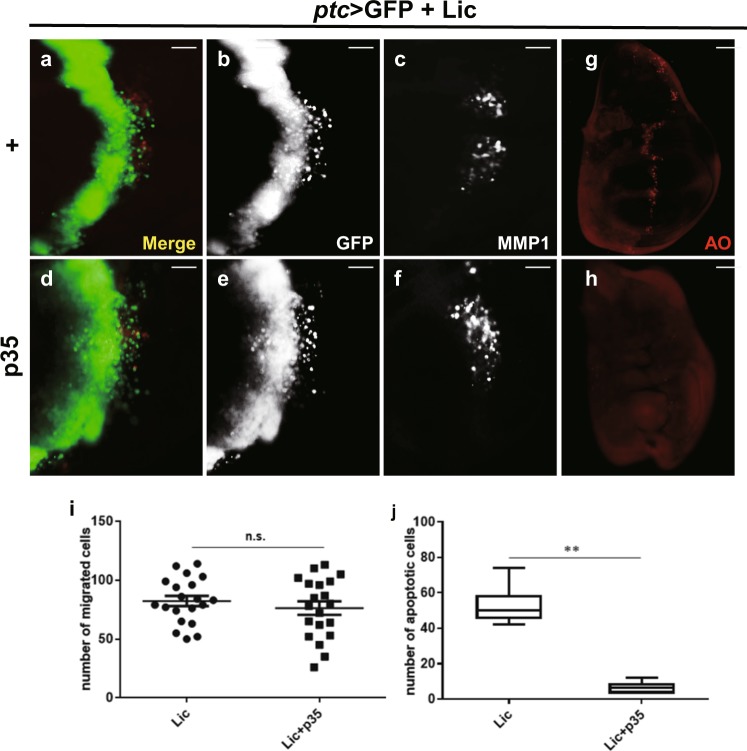
Lic-induced cell invasion is independent of cell death. Fluorescent micrographs of third instar wing discs are shown (**a**–**h**). *ptc* > Lic induced cell death (**g**), but not cell migration (**a**, **b**) and MMP1 activation (**c**), was inhibited by p35 overexpression (**d**–**f**, **h**). Scale bars, 20 μm. The statistical analysis of the number of migrated cells and apoptotic cells are shown in (**i**, **j**). One-way ANOVA test was used to calculate statistical significance, *n* ≥ 15, mean + s.d., n.s., *P* > 0.05; **P < 0.01

Previous studies have shown that JNK pathway activation promotes cell invasion in a context-dependent manner, but cell death in a non-tissue-specific manner. For instance, activation of JNK along the A/P compartment boundary in wing discs by *ptc* > Hep triggers both cell death and invasion (Supplementary Figure 12f, g and j), while *GMR* > Hep only induces cell death, but not cell invasion in eye discs (Supplementary Figure 12a, b and e). Consistently, we found that Lic promotes JNK-mediated cell invasion in a tissue-specific manner, and cell death non-tissue specifically (Supplementary Figure 12c, d, h and i). These data suggest cell death and cell invasion are independent outcomes of JNK activation. Consistent with this notion, expression of the baculovirus protein p35, which has been reported to inhibit programmed cell death in *Drosophila*^48,49^, fully suppressed *ptc* > Lic-triggered cell death (Fig. 7g, h, j), but had no effect on cell invasion and MMP1 activation (Fig. 7d–f, i). Taken together, the above results suggest that Lic-induced cell invasion is a direct outcome of JNK activation, but not a secondary effect of cell death.

## Discussion

*lic* encodes the *Drosophila* MKK3 ortholog that has been previously characterized as the MAP kinase kinase regulating p38 signaling in cell growth, stress response, innate immunity and asymmetric egg development in oogenesis^12^. In this study, we provide the first in vivo evidence that Lic acts as an essential regulator of the JNK signaling crucial for invasive cell migration triggered by the loss-of-cell polarity in the wing epithelia and oncogenic cooperation induced eye tumor invasion to the VNC. Furthermore, Lic regulates physiological JNK-dependent cell migration in thorax development. Finally, these functions of Lic are evolutionarily conserved as human MKK3 appears to work similarly in flies. Interestingly, MKK3 has previously been implicated in tumor cell invasion in vitro^37,50^, yet this function has not been confirmed in vivo, and its underlying mechanism remains elusive. Thus, our results not only shed light on the mechanism of MKK3-mediated tumor cell invasion, but also provide potential therapeutic strategies for MKK3-related cancer treatment.

Although previous studies have characterized Lic as a MAPKK for the p38 kinase, our data suggest that Lic regulates JNK signaling in cell invasion via a mechanism that is independent of p38. Firstly, loss of *lic*, but not *p38*, suppressed loss-of-cell polarity-triggered JNK-dependent cell invasion (Fig. 1l, o, q and data not shown). Secondly, ectopic Lic-triggered JNK activation and cell invasion were not affected by loss of p38 (Supplementary Figure 8a–f). Thirdly, Lic acts in parallel with Hep, as depletion of *lic* did not suppress ectopic Hep-triggered cell invasion (Fig. 5e, f, n), and vice versa (Fig. 5a, b and Supplementary Figure 11a, b and e). It is plausible that Lic acts as another JNK kinase that performs redundant function with Hep (Supplementary Figure 11f), since co-expression of Lic and Hep displays synergistic effect in promoting cell invasion (Fig. 5g, n), and Egr-triggered cell invasion is only partially suppressed in heterozygous *hep* or *lic* mutants, but fully blocked in transheterozygous double mutants (Fig. 5i–l, o).

A close relationship between cell death and migration has been reported^51–53^, and under certain situations dying cells could cause compensatory proliferation and subsequent migration of surrounding cells^54–56^. Consistent with the role of JNK signaling in cell death, ectopic expression of Lic triggers not only EMT-like cell invasion, but also cell death marked by acridine orange (AO) staining (Fig. 7a–c and g). Blocking cell death by expressing the baculovirus protein p35 effectively abolished Lic-induced cell death, but does not affect Lic-initiated cell invasion and MMP1 activation (Fig. 7d–f, h–j), suggesting Lic-triggered cell invasion is a direct outcome of JNK signaling activation, but not a secondary effect caused by cell death.

## Materials and methods

### *Drosophila* stocks and genetics

Stocks were raised on standard *Drosophila* media and crosses were performed at 25 °C, cell migration assay were performed at 29 °C. Following fly stocks have been described previously: *w*^*1118*^^4^; *TRE-RFP*^28^; *GMR*-Gal4, *ey*-Gal4, *pnr*-Gal4, *ptc*-Gal4, *UAS-Ras*^*V12*^, *lgl*^*4*^, *UAS*-Hep^WT^^20^; *UAS*-GFP, *UAS*-p35, *UAS*-Bsk^DN^, *UAS*-Puc, *puc*^*E69*^, *UAS*-*scrib-RNAi*^18^; *GFP-RNAi*^57^; *UAS*-Lic^14^ *hep*^*1*^^58^; *bsk*^*1*^^59^; *lic*^*G0252*^^60^; *UAS*-Egr^Regg1^^61^ and *UAS*-Hep^CA^^62^. Strains obtained from the Bloomington Drosophila Stock Center (BDSC) are *hs*-Gal4 (#1799), *UAS*-LacZ (#3956), *UAS*-*p38b-RNAi* (#29405), *UAS-lic-RNAi* (#31643), *UAS*-p38b^DN^ (#59005), *UAS-hep-RNAi* (#28710), *UAS-dTAK1-RNAi* (#31045) and *UAS-wnd-RNAi* (#27525). Strains received from the Vienna Drosophila RNAi Center (VDRC) are *UAS-lic-RNAi* (#20166); *UAS-wnd-RNAi* (#13786) and *UAS-hep-RNAi* (#26929). *UAS-dTAK1-RNAi* (#1388R-2) is acquired from the National Institute of Genetics (NIG-FLY). Fluorescently labeled invasive tumors were produced by the following strains: *y, w, ey*-Flp; *tub*-Gal80, FRT40A; *Act* > *y+* > Gal4, *UAS*-GFP (40A tester), *lgl*^4^ FRT40A *UAS*-*Ras*^*V12*^ (40A tester) and *ey*-Flp, *Act* > *y+* > Gal4, *UAS*-GFP^63^. *UAS*-Lic^KD^ were gifts from Prof. Haiyun Song.

### *UAS*-MKK3 transgenic flies

The MKK3 coding region was amplified by RT-PCR from 293 cell line, and analyzed by standard sequencing. The p*UAST*-MKK3 construct was introduced into the germ line by injection and *UAS*-MKK3 transgenic lines were established by standard genetics.

### Antibodies

The following primary antibodies were used: mouse anti-MMP1 (1:200), rabbit anti-phospho-JNK (1:200, Calbiochem, San Diego, CA, USA) and mouse-anti-β-Gal (1:500, DSHB). The following secondary antibodies were used: anti-mouse CY3 (1:1000, CST) and anti- rabbit CY3 (1:1000, CST). The following primary antibodies were used for western blot analysis: rabbit anti-p-JNK (9251S, 1:1,000, Cell Signaling Technology), Rabbit anti-JNK (sc-7345, 1:500, Santa Cruz Biotechnology).

### X-gal staining

Eye and wing discs were dissected from third instar larvae in PBST (1× PBS pH 7.0, 0.1% Triton X-100) and stained for β-galactosidase activity as described^64^.

### AO staining

Eye and wing discs were dissected from 3rd instar larvae in 1× PBS (phosphate-buffered saline) and incubated in 1 × 10^−5^ M acridine orange (AO) for 5 min at room temperature^65^.

### Imaging of fly eyes and wings

Three-day-old flies were collected and frozen at −80 °C. When taking pictures, flies were unfrozen at room temperature and placed on 1% agarose plate. Light images of eye was taken by OLYMPUS stereo microscope SZX16. Wings were dissected and placed on slide with alcohol/glycerol (1:1) buffer. Light images of wing were taken by OLYMPUS BX51 microscope.

### qRT-PCR

Total RNA was extracted from flies’ cephalosome of indicated genotype using PureLink RNA Mini Kit (Ambion). Total RNA was reverse-transcribed into cDNA with the PrimeScript RT Master Mix (Takara), quantitative PCR was performed with SYBR Premix ExTaq II (Takara) and quantified by the Stratagene MX3000P system (Stratagene). RP49 was used as an internal control. The following primers were used for real-time PCR.

*lic*, sense primer: 5′-GGCCGCTACCCATACGACAA-3′;

*lic*, antisense primer: 5′-ACTGTCCTCAACCACCTGA-3′.

*p38b*, sense primer: 5′-GAAGCGCACCTATCGGGAAC-3′;

*p38b*, antisense primer: 5′-GACATCCAGCAGACCAATAA-3′.

*Rp49*, sense primer: 5′-CCACCAGTCGGATCGATATGC-3′;

*Rp49*, antisense primer: 5′-CTCTTGAGAACGCAGGCGACC-3′.

## Supplementary information

Supplementary Information
