# Supplementary material for: MKK3 modulates JNK-dependent cell migration and invasion
Source: Cell Death Dis. 2019 Feb 15;10(3):149. doi: 10.1038/s41419-019-1350-6 (PMC6377636; doi:10.1038/s41419-019-1350-6)
Supplement: Supplementary file 1 — Supplementary Information [file 41419_2019_1350_MOESM1_ESM.docx]

**Supplementary Information**

**Genotypes of flies used in article:**

**Figure 1**

(a, b, c, d) *ptc*-Gal4, *UAS*-GFP/+

(e, f, g) *ptc*-Gal4, *UAS*-GFP, *UAS*-*scrib*-IR/*+*

(h, i, j) *ptc*-Gal4, *UAS*-GFP, *UAS*-*scrib*-IR/+; *UAS*-Puc/+

(k, l, m) *ptc*-Gal4, *UAS*-GFP, *UAS*-*scrib*-IR/*UAS*-*lic-IR^V20166^*

(n, o, p) *ptc*-Gal4, *UAS*-GFP, *UAS*-*scrib*-IR/+; *UAS*-*lic-IR^BL31643^*/+

**Figure 2**

(a, b, c, e) *ptc*-Gal4, *UAS*-GFP/+

(d) *ptc*-Gal4, *UAS*-GFP, *TRE-RFP*/+

(f) *ptc*-Gal4/+; *puc*-LacZ/*UAS*-GFP

(g, h, i, k) *ptc*-Gal4, *UAS*-GFP/+; *UAS*-Lic/+

(j) *ptc*-Gal4, *UAS*-GFP, *TRE-RFP*/+; *UAS*-Lic/+

(l) *ptc*-Gal4/+; *puc*-LacZ/*UAS*-Lic

(m, n, o, q) *ptc*-Gal4, *UAS*-GFP/+; *UAS*-Puc/*UAS*-Lic

(p) *ptc*-Gal4, *UAS*-GFP, *TRE-RFP*/+; *UAS*-Puc/*UAS*-Lic

(r) *ptc*-Gal4/+; *puc*-LacZ/*UAS*-Puc*, UAS*-Lic

**Figure 3**

(a, b, c, e) *ptc*-Gal4, *UAS*-GFP/+

(d) *ptc*-Gal4, *UAS*-GFP, *TRE-RFP*/+

(f) *ptc*-Gal4/+; *puc*-LacZ/*UAS*-GFP

(g, h, i, k) *ptc*-Gal4, *UAS*-GFP/+; *UAS*-MKK3/+

(j) *ptc*-Gal4, *UAS*-GFP, *TRE-RFP*/+; *UAS*-MKK3/+

(l) *ptc*-Gal4/+; *puc*-LacZ/*UAS*-MKK3

(m, n, o, q) *ptc*-Gal4, *UAS*-GFP/+; *UAS*-Puc/*UAS*-MKK3

(p) *ptc*-Gal4, *UAS*-GFP, *TRE-RFP*/+; *UAS*-Puc/*UAS*-MKK3

(r) *ptc*-Gal4/+; *puc*-LacZ/*UAS*-Puc*, UAS*-MKK3

**Figure 4**

(a, e) *ey-*FLP/+; *tub*-Gal80^ts^, FRT40A/*lgl^4^* FRT40A *UAS*-Ras^V12^; *act>y+>*Gal4, *UAS*-GFP/*UAS*-GFP

(b, f) *ey-*FLP/+; *tub*-Gal80^ts^, FRT40A/*lgl^4^* FRT40A *UAS*-Ras^V12^; *act>y+>*Gal4, *UAS*-GFP/*UAS*-Puc

(c, g) *ey-*FLP/+; *tub*-Gal80^ts^, FRT40A/*lgl^4^* FRT40A *UAS*-Ras^V12^; *act>y+>*Gal4, *UAS*-GFP/*UAS*-*lic-IR^BL31643^*

(d, h) *ey-*FLP/*lic^G0252^*; *tub*-Gal80^ts^, FRT40A/*lgl^4^* FRT40A *UAS*-Ras^V12^; *act>y+>*Gal4, *UAS*-GFP/+

(j, n) *ey-*Flp, *act>y+>*Gal4, *UAS*-GFP/+; *UAS*-Lic/+

(k, o) *ey-*Flp, *act>y+>*Gal4, *UAS*-GFP/+; *UAS*-Ras^V12^/+

(l, p) *ey-*Flp, *act>y+>*Gal4, *UAS*-GFP/+; *UAS*-Ras^V12^/*UAS*-Lic

(m, q) *ey-*Flp, *act>y+>*Gal4, *UAS*-GFP/+; *UAS*-Ras^V12^/*UAS*-MKK3

**Figure 5**

(a) *ptc*-Gal4, *UAS*-GFP/+; *UAS*-Lic/*UAS*-GFP

(b) *ptc*-Gal4, *UAS*-GFP/+; *UAS*-*hep-IR^V26929^*/*UAS*-Lic

(c) *ptc*-Gal4, *UAS*-GFP/*UAS*-*dTAK1-IR^NIG1388R-2^*; *UAS*-Lic/+

(d) *ptc*-Gal4, *UAS*-GFP/+; *UAS*-Lic/*UAS-wnd-IR^V13786^*

(e) *ptc*-Gal4, *UAS*-GFP/*UAS*-Hep^WT^; *UAS*-GFP/+

(f) *ptc*-Gal4, *UAS*-GFP/*UAS*-Hep^WT^, *UAS*-*lic-IR^V20166^*

(g) *ptc*-Gal4, *UAS*-GFP/*UAS*-Hep^WT^; *UAS*-Lic/+

(h) *ptc*-Gal4, *UAS*-GFP/*UAS*-Hep^WT^; *UAS*-MKK3/+

(i) *ptc*-Gal4*, UAS-*Egr, *tublin*-Gal80^ts^/+; *UAS*-GFP/+

(j) *hep^1^*/+; *ptc*-Gal4*, UAS-*Egr, *tublin*-Gal80^ts^/+; *UAS*-GFP/+

(k) *lic^G0252^*/+; *ptc*-Gal4*, UAS-*Egr, *tublin*-Gal80^ts^/+; *UAS*-GFP/+

(l) *lic^G0252^*/*hep^1^*; *ptc*-Gal4*, UAS-*Egr, *tublin*-Gal80^ts^/+; *UAS*-GFP/+

**Figure 6**

(a) *pnr*-Gal4/*puc-LacZ*

(b) *UAS*-*lic-IR^V20166^*/+; *pnr*-Gal4/*puc-LacZ*

(c) *pnr*-Gal4/+

(d) *UAS*-*lic-IR^V20166^*/+; *pnr*-Gal4/+

(e) *bsk^1^*/+; *pnr-Gal4*/+

(f) *bsk^1^*/*UAS*-*lic-IR^V20166^*; *pnr*-Gal4/+

(g) *pnr*-Gal4/*puc^E69^*

(h) *UAS*-*lic-IR^V20166^*/+; *pnr*-Gal4/*puc^E69^*

(i) *pnr*-Gal4/*UAS*-MKK3

(j) *UAS*-*lic-IR^V20166^*/+; *pnr*-Gal4/UAS-MKK3

**Figure 7**

(a, b, c, g) *ptc*-Gal4, *UAS*-GFP/+; *UAS*-Lic/+

(d, e, f, h) *ptc*-Gal4, *UAS*-GFP/+; *UAS*-Lic/*UAS*-p35

**Supplementary Figure 1**

(a) *ptc*-Gal4, *UAS*-GFP, *UAS*-*scrib*-IR/+; *UAS*-LacZ/+

**Supplementary Figure 2**

(a) *ptc*-Gal4, *UAS*-GFP, *UAS*-*scrib*-IR/*TRE-RFP*; *UAS*-LacZ/+

(b, c) *ptc*-Gal4, *UAS*-GFP, *UAS*-*scrib*-IR/+; *UAS*-LacZ/+

(d) *ptc*-Gal4, *UAS*-GFP, *UAS*-*scrib*-IR/*+*; *puc*-LacZ/*UAS*-LacZ

(e) *ptc*-Gal4, *UAS*-GFP, *UAS*-*scrib*-IR/*TRE-RFP*; *UAS*-*lic-IR^BL31643^*/+

(f, g) *ptc*-Gal4, *UAS*-GFP, *UAS*-*scrib*-IR/+; *UAS*-*lic-IR^BL31643^*/+

(h) *ptc*-Gal4, *UAS*-GFP, *UAS*-*scrib*-IR/*+*; *puc*-LacZ/*UAS*-*lic-IR^BL31643^*

**Supplementary Figure 3**

(a) *GMR*-Gal4/+

(b) *GMR*-Gal4/*UAS*-*lic-IR^V20166^*

(c) *GMR*-Gal4/+; *UAS*-*lic-IR^BL31643^*/+

**Supplementary Figure 4**

(a-c) *ptc*-Gal4, *UAS*-GFP/+; *UAS*-Lic/*UAS*-LacZ

(d-f) *ptc*-Gal4, *UAS*-GFP/+; *UAS*-Lic/*UAS*-Bsk^DN^

**Supplementary Figure 5**

(a, b) *ptc*-Gal4, *UAS*-GFP, *TRE-RFP*/+; *UAS*-LacZ/+

(c-g) *ptc*-Gal4, *UAS*-GFP, *TRE-RFP*/+; *UAS*-Lic/+

**Supplementary Figure 7**

(a-d) *ptc*-Gal4, *UAS*-GFP/+; *UAS*-Lic^KD^/

**Supplementary Figure 8**

(a, b, c) *ptc*-Gal4, *UAS*-GFP, *TRE-RFP*/+; *UAS*-Lic/*UAS-p38b-IR^BL29405^*

(d, e, f) *UAS*-p38^DN^; *ptc*-Gal4, *UAS*-GFP, *TRE-RFP*/*+*; *UAS*-Lic/*+*

**Supplementary Figure 9**

(a, b) *ptc*-Gal4, *UAS*-GFP/+; *UAS*-MKK3/*UAS*-LacZ

(c) *ptc*-Gal4, *UAS*-GFP, *TRE-RFP*/+; *UAS*-MKK3/*UAS*-LacZ

(d, e) *ptc*-Gal4, *UAS*-GFP/+; *UAS*-MKK3/*UAS*-Bsk^DN^

(f) *ptc*-Gal4, *UAS*-GFP, *TRE-RFP*/+; *UAS*-MKK3/*UAS*-Bsk^DN^

**Supplementary Figure 10**

(a, b, c) *ptc*-Gal4, *UAS*-GFP, *TRE-RFP*/+; *UAS*-MKK3/*UAS-p38b-IR ^BL29405^*

(d, e, f) *UAS*-p38^DN^; *ptc*-Gal4, *UAS*-GFP, *TRE-RFP*/*+*; *UAS*-MKK3/*+*

**Supplementary Figure 11**

(a) *ptc*-Gal4, *UAS*-GFP/+; *UAS*-Lic/*UAS*-GFP

(b) *ptc*-Gal4, *UAS*-GFP/+; *UAS*-Lic/*UAS-hep-RNAi^BL28710^*

(c) *ptc*-Gal4, *UAS*-GFP/+; *UAS*-Lic/*UAS-dTAK1-RNAi^BL31045^*

(d) *ptc*-Gal4, *UAS*-GFP/+; *UAS*-Lic/*UAS-wnd-RNAi^BL27525^*

**Supplementary Figure 12**

(a) *GMR*-Gal4, *UAS*-Hep/*TRE-RFP*; *UAS*-GFP/+

(b) *GMR*-Gal4, *UAS*-Hep/*+*; *UAS*-GFP/+

(c) *GMR*-Gal4*, TRE-RFP*/+; *UAS*-GFP/*UAS*-Lic

(d) *GMR*-Gal4/*+*; *UAS*-GFP/*UAS*-Lic

(f) *ptc*-Gal4, *UAS*-GFP, *TRE-RFP*/*UAS*-Hep

(g) *ptc*-Gal4, *UAS*-Hep/*+*; *UAS*-GFP/+

(h) *ptc*-Gal4, *UAS*-GFP, *TRE-RFP*/+; *UAS*-Lic/+

(i) *ptc*-Gal4/*+*; *UAS*-GFP/*UAS*-Lic

**Supplementary Figure**


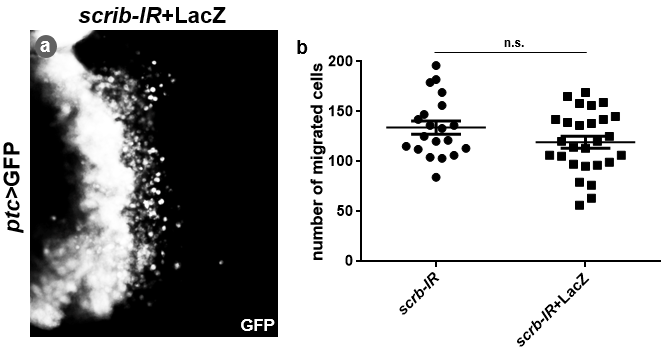


**Supplementary Figure 1 *ptc>scrib-RNAi* induced cell migration was not affected by a *UAS*-LacZ transgene.**

Fluorescent micrograph of a third instar wing disc is shown (a). Depletion-of-*scrib* induced cell migration was not affected by the addition of a *UAS*-LacZ transgene (a). The number of migrated cells were quantified and shown in (b), and Student-t test was used to calculate statistical significance, n≥20, mean + s.d., n.s., *P*>0.05


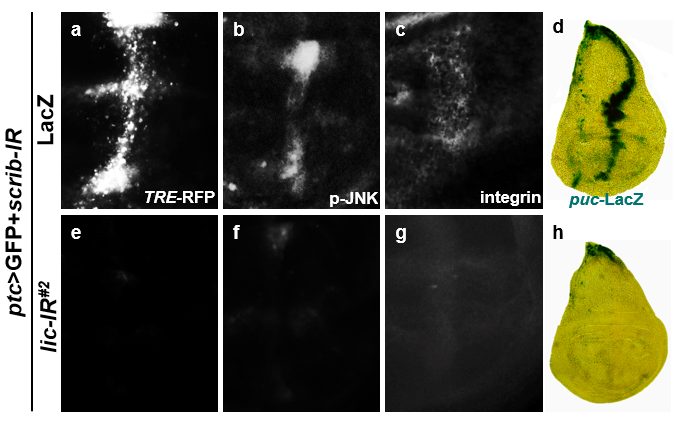


**Supplementary Figure 2 Loss of *lic* impeded *scrib-RNAi*-induced JNK activation and cell migration.**

Fluorescent (a-c, e-g) and light (d, h) micrographs of third instar wing discs are shown. *ptc>scrib-IR* induced activation of *TRE-RFP*, p-JNK, integrin and *puc*-LacZ were not affect by expressing LacZ (a-d), but were significantly suppressed by expressing a *lic-RNAi* (e-h).


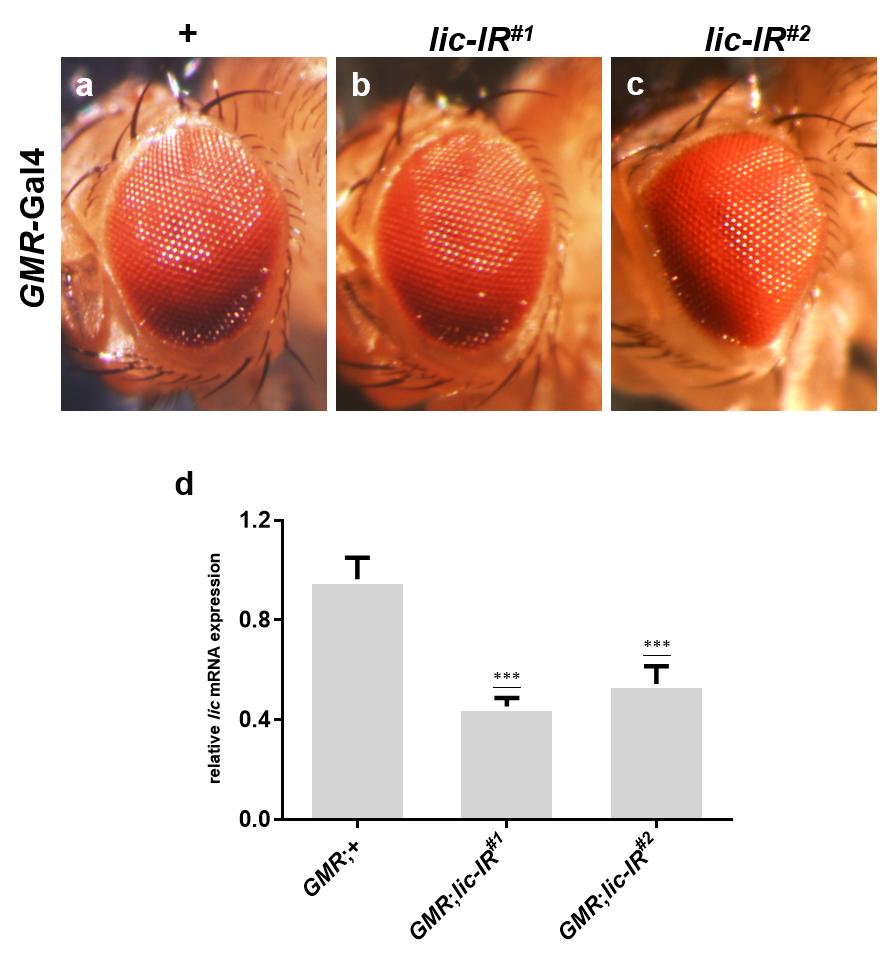


**Supplementary Figure 3 Verification of two independent *UAS-lic-IR* transgenes.** Light micrographs of *Drosophila* adult eyes (a, b, c) are shown. Compared with the *GMR*-Gal4 control (a), overexpressing *lic-IR^#1^* or *lic-IR^#2^* produced no obvious phenotype (b, c). Scale bars, 100 μm. qRT-PCR analysis indicates that *lic* transcription in adult eyes is significantly reduced by the expression of two *lic-IR* (d). Student-t test was used to calculate statistical significance, mean + s.d., ****P* < 0.001.


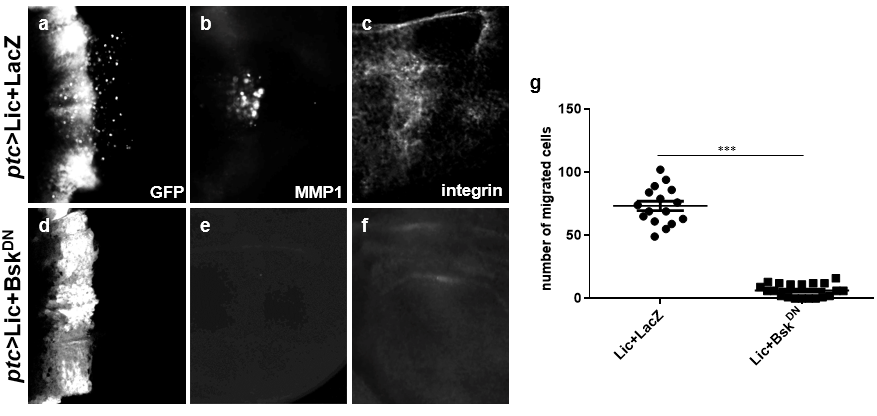


**Supplementary Figure 4 Lic activates JNK-dependent cell migration.**

Fluorescent micrographs of third instar wing discs (a-f) are shown. Expression of Bsk^DN^ (d-f), but not LacZ (a-c) suppressed Lic-triggered cell migration (d), MMP1 (e) and integrin (f) activation. Statistics of migrated cell numbers was shown in (g), and One-way ANOVA test was used to calculate statistical significance, n≥15, mean + s.d., ***, *P*<0.001.


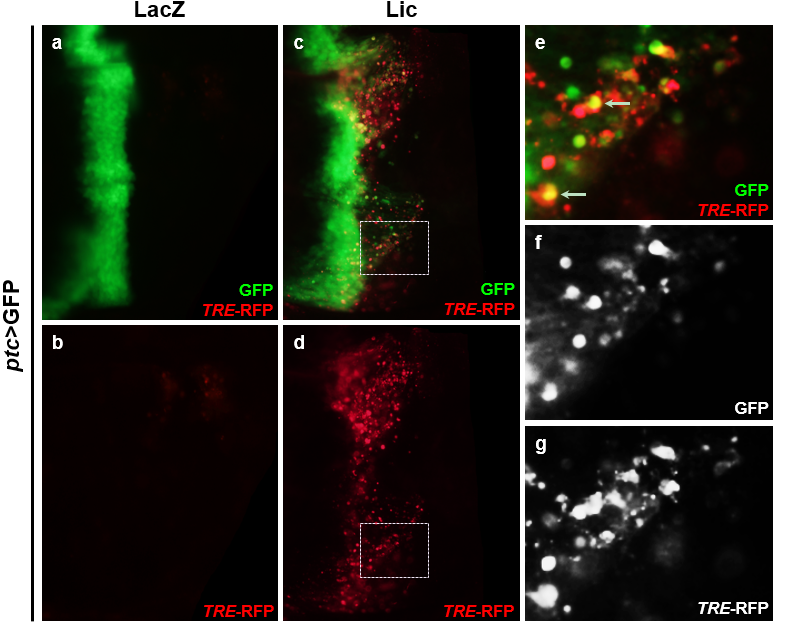


**Supplementary Figure 5 Lic triggers autonomous and non-autonomous JNK activation.**

Fluorescent micrographs of third instar wing discs are shown. Compared with the control (a, b), Lic expression driven by *ptc*-Gal4 promoted cell migration and *TRE-RFP* activation (c, d). (e-g) are higher magnifications of the dashed box regions in c and d, and white arrows indicate cells with coincidence of GFP channel and RFP channel.

**
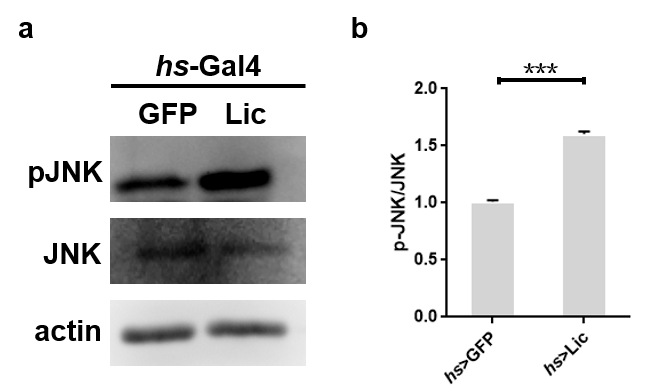
**

**Supplementary Figure 6 Overexpression of Lic promotes JNK phosphorylation**

Ectopic Lic up-regulates the phosphorylation level of JNK as shown by Western blot (a). The phosphorylation levels of JNK are normalized to the total JNK levels (b). Statistical significance is determined with Student’s t test, ***, *P*< 0.001.


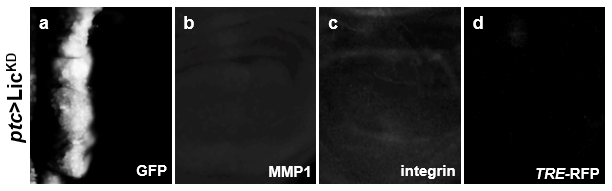


**Supplementary Figure 7 Lic^KD^ failed to induce invasive cell migration.**

Fluorescent micrographs of third instar wing discs are shown. Expression of a kinase dead form of Lic (Lic^KD^) failed to trigger cell migration (a), MMP1 induction (b), integrin accumulation (c) and *TRE-RFP* activation (d).


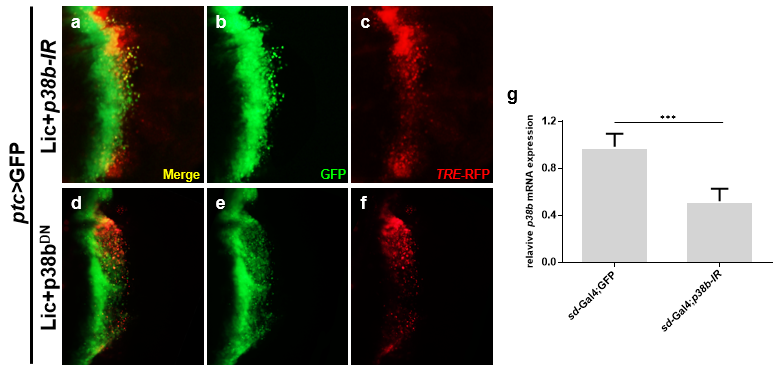


**Supplementary Figure 8** **Lic induces p38-independent JNK activation and cell migration****.**

Fluorescent micrographs of third instar wing discs (a-f) are shown. Lic induced cell invasion and *TRE-RFP* activation cannot be suppressed by co-expression of *p38b-IR* (a-c), or p38b^DN^ (d-f). qRT-PCR analysis indicates that *p38b* transcription in wing disc is significantly reduced by *sd>p38b-IR* (g). Student-t test was used to calculate statistical significance, mean + s.d., ****P* < 0.001.


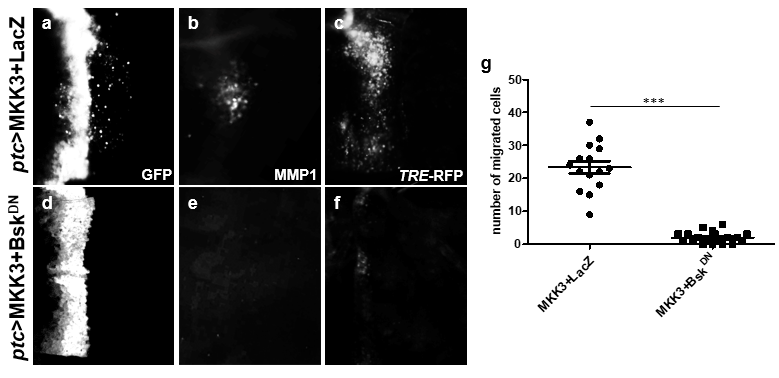


**Supplementary Figure 9 MKK3 activates JNK-dependent cell migration.**

MKK3-induced cell migration, MMP1 accumulation, and *TRE-RFP* activation were suppressed by co-expression of Bsk^DN^ (d-f). *UAS*-LacZ was included (a-c) as a negative control to demonstrate that the suppression is not a result of Gal4 titration by another UAS line. Statistics of migrated cell numbers was shown in (g), and One-way ANOVA test was used to calculate statistical significance, n≥10, mean + s.d., ***, *P*<0.001.


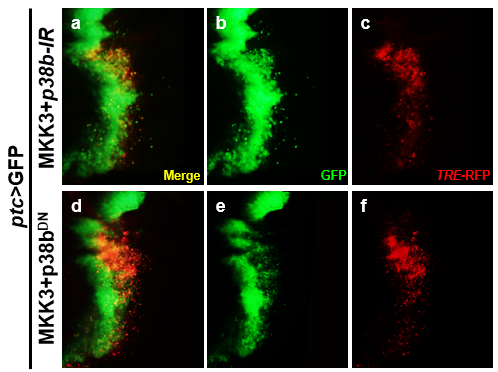


**Supplementary Figure 10** **MKK3 promotes p38-independent JNK activation and cell migration.**

Fluorescent micrographs of third instar wing discs are shown. Overexpression of MKK3-induced cell migration and JNK activation were not suppressed by expressing *p38b-IR* (a-c) or p38^DN^ (d-f).


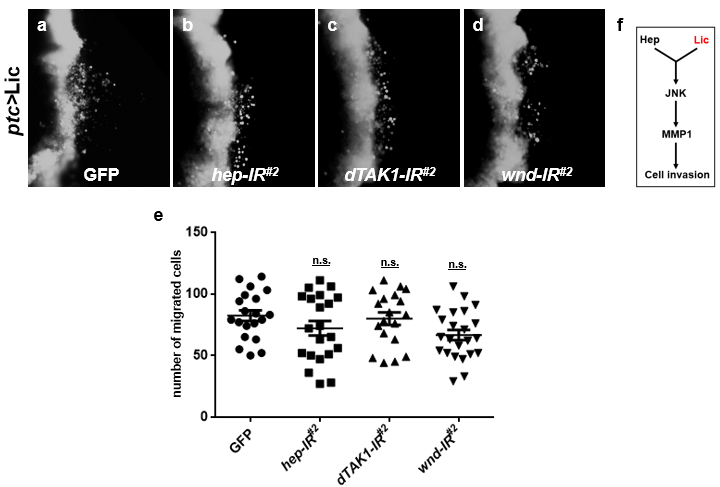


**Supplementary Figure 11** **Lic acts in parallel with Hep to promote JNK-mediated cell invasion.**

Compared with the control (a), *ptc*>Lic-induced cell migration remained unaffected by knockdown of *hep-IR* (b), *dTAK1-IR* (c) or *wnd-IR* (d). The number of migrated cells were quantified and shown in (e), and One-way ANOVA test was used to calculate statistical significance, n≥15, mean + s.d., n.s., *P*>0.05. A schematic diagram of Lic-induced JNK-dependent cell invasion was shown in (f).

**
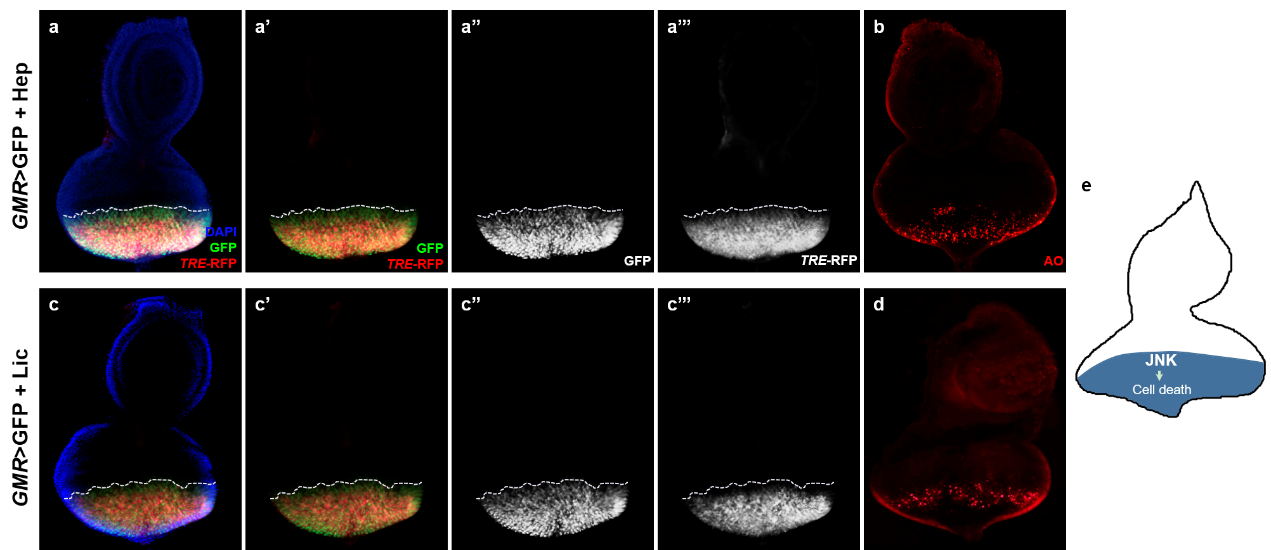
**

**
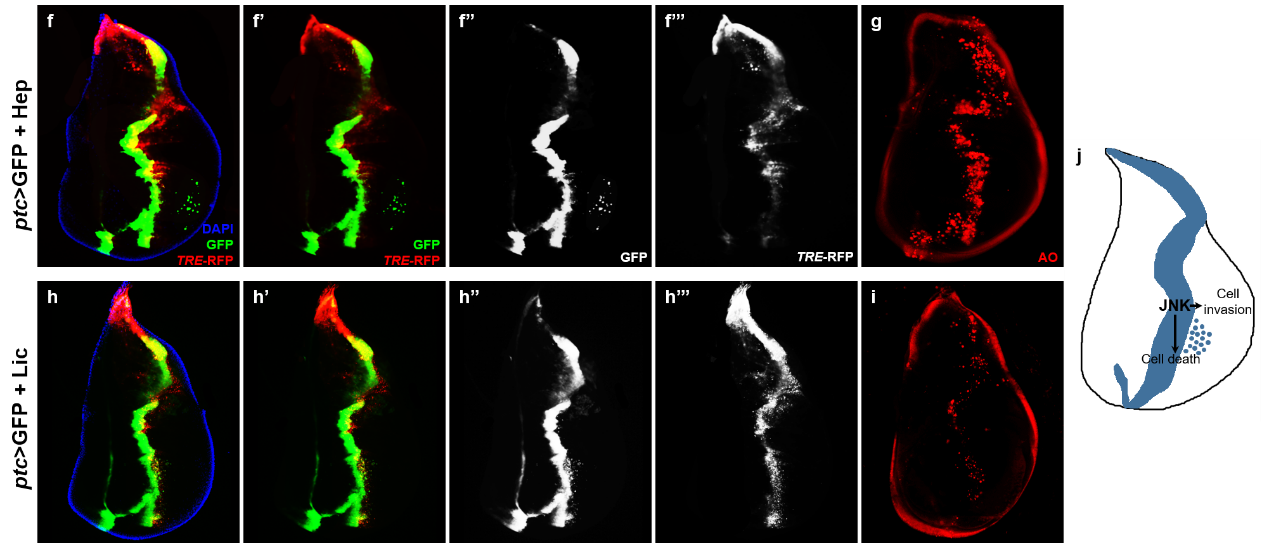
**

**Supplementary Figure 12 Lic modulates JNK-dependent cell invasion in a tissue-specific manner**

Fluorescent micrographs of third instar eye (a-d) and wing discs (f-i) are shown. In eye discs, expression of Hep or Lic driven by *GMR*-Gal4 induced JNK activation (a, c) and cell death (b, d), but no cell migration (a, c). Expression of Hep or Lic driven by *ptc*-Gal4 along the A/P boundary in wing discs triggered JNK activation (f, h), cell death (g, i) and cell invasion (f, h). (e) and (j) are schematic diagrams of JNK-induced cell death and/or cell invasion in different tissues.
